# “Lock to Live” for firearm and medication safety: Feasibility and acceptability of a suicide prevention tool in a learning healthcare system

**DOI:** 10.3389/fdgth.2022.974153

**Published:** 2022-09-06

**Authors:** Jennifer M. Boggs, LeeAnn M. Quintana, Arne Beck, Samuel Clinch, Laura Richardson, Amy Conley, Julie E. Richards, Marian E. Betz

**Affiliations:** ^1^ Kaiser Permanente Colorado Institute for Health Research, Aurora, CO, United States; ^2^ Kaiser Permanente Colorado Behavioral Health Department, Denver, CO, United States; ^3^ Kaiser Permanente Washington Heath Research Institute, Seattle, WA, United States; ^4^ Department of Emergency Medicine, University of Colorado School of Medicine, Aurora, CO, United States

**Keywords:** web-based, firearms, lethal means, medication access, digital health, Lock to Live

## Abstract

**Objective:**

Few patients with suicide risk are counseled on lethal means safety by health providers. This study tested the feasibility of different delivery methods for Lock to Live (L2L), a web-based decision aid of safe storage options for firearms and medications.

**Methods:**

Patients reporting suicide ideation on the PHQ9 depression screener during outpatient health visits were included. Invitation messages to visit L2L were sent *via* combinations of email, text, Electronic Health Record (EHR) message, mailed letter, or provider referral, followed by a survey about storage behavior and acceptability. Provider interviews evaluated logistical considerations and acceptability.

**Results:**

The population-based method reached 2,729 patients and the best method (EHR message plus 2 email reminders) had 11% uptake (L2L visitation rate). Provider referral had small reach (14 patients) and 100% uptake (all visited). Provider interviews identified several strategies to promote uptake including: EHR reminders, provider training, quality metrics with accountability, a clearly communicated lethal means screening/counseling policy, and strong organizational leadership support.

**Conclusion:**

Despite the low uptake for population-based (11%), far more patients with suicide risk were engaged in the L2L tool through population-based outreach than provider-referral over the same time frame.

## Introduction

Reducing access to lethal means for suicide is highly effective for suicide prevention (1–3). In the United States, this involves targeting the most common lethal means—securing firearms and mitigating risk for overdose (e.g., disposing of unused medications or asking a support person to dispense prescription medications during high-risk times) (2). While many countries rely on legal interventions to address firearm safety broadly (e.g., for safe storage, training for owners, and mental health related restrictions on access) (1), there are considerable political roadblocks to firearm legislation in the United States. Therefore, the health care setting has become an important avenue to deliver public health interventions to increase firearm safety for patients at risk of suicide (4). Lethal means counseling is recommended (5) and involves reducing access to common lethal means during suicidal crises. However, many patients with suicide risk are not counseled on lethal means safety across health settings (6), indicating the need for new approaches to address this gap.

There are several factors contributing to low rates of lethal means counseling particularly within emergency medicine and behavioral health, which include: time-constraints, providers’ lack of perceived benefit for preventing suicide (among emergency medicine providers), lack of training or knowledge of safe storage options, and fear that asking about firearms will damage the patient/clinician relationship (7, 8). Population-based interventions that don't rely on providers can address these challenges and be broadly disseminated because they are typically lower-cost, particularly at scale. Caring letters are an example of a low-intensity population-based intervention resulting in reduction in suicidal behavior (9).

One challenge to expanding firearm safety interventions delivered by clinicians is whether patients disclose access to firearms. There is disagreement in the literature on this point. Some surveys of firearm owners suggest that most are receptive and understand the importance within the context of suicide prevention (10, 11). Other studies show that firearm owners tend to have increased concerns around privacy, both in general and specifically as it relates to recording firearm ownership in medical records (12–16). Education on safe storage delivered anonymously may be more acceptable to firearm owners.

To address these challenges, we tested a population-based approach using an anonymous, web-based, self-administered decision aid for safe firearm and medication storage in patients with suicide risk called Lock to Live (http://lock2live.org/) (17). *Lock to Live* (L2L) was designed with input from over 60 stakeholders, including patients with lived experience (18), medical and mental health providers and firearm owners. Decision aids have been shown to effectively facilitate health decisions that are complicated by first providing education, then options to patients that incorporate their values and preferences into the decision-making process. L2L is based on the Ottawa's Decision Support Framework (19), which posits that the quality of decisions is higher when people are knowledgeable, understand the conflict or uncertainty points, and have identified and incorporated their own values. The behavioral economics concept of “nudges” is also incorporated by presenting effort-laden storage options alongside easy ones to subtly persuade patients to choose the easy options (18). L2L is anonymous (no patient identifiers are entered into the tool) and focuses on voluntary, temporary reductions in access; it provides information on costs for both in-home and out-of-home storage options, whether a background check is required to return firearms, and medication management options that address the need for access to daily medications. L2L has information on what types of storage options require background checks to encourage compliance with state background check laws for transfers between non-family members.

Initial testing of L2L with a tablet in an emergency department setting showed that it was highly acceptable across a broad range of provider roles (physicians, nurses, social workers). L2L addresses the lack of provider knowledge for different firearm and medication storage options (20). A survey showed that clinicians thought L2L would help them counsel suicidal adult patients on safe firearm storage options (21). A small pilot trial of 49 adult patients, evaluated in the emergency setting for a suicide-related concern, showed high satisfaction with L2L and non-significant increases in safe storage behavior (22). Enrollment was low—likely due to privacy concerns about firearm access alongside a high level of suicide risk and vulnerability in the emergency setting. These pilot findings support the need to pilot test new implementation strategies that address concerns about privacy and reach greater volumes of patients. Importantly, we recognize the need for more effectiveness data on the Lock to Live tool. However, the implementation strategy needs to be tested first because it would be paired with the tool when tested in a future larger trial.

The purpose of this study was to test the feasibility (reach and uptake) and acceptability (patient survey and provider interviews) of delivering the L2L web-based decision aid for safe firearm storage using population-based and provider-referral outreach in the *outpatient setting*. Reach was defined as the number of patients outreached and uptake was the number of patients who visited L2L. An anonymous follow-up survey evaluated impacts on storage behavior and patient acceptability. Provider interviews evaluated logistical considerations and acceptability.

## Methods

### Setting

The sample included adult members of a large integrated health care system (Kaiser Permanente Colorado) who reported any frequency of suicidal thoughts on the ninth question of the Patient Health Questionnaire (PHQ-9) (23) from October 2019 to April 2020. The ninth question asked whether patients have had “thoughts that you would be better off dead, or thoughts of hurting yourself in some way” in the past 2 weeks. Response options include: not at all (0), several days (1), more than half the days (2), or nearly every day (3). The PHQ-9 was administered on a Tablet in the waiting room prior to all mental health visits and on paper for selected medical visits where patients are routinely screened or treated for depression and/or anxiety.

### Study design

We used a mixed-methods (QUAN qual) embedded design (24) to assess the feasibility and acceptability of different outreach methods for L2L *via* different combinations of population-based outreach methods [email, text, Electronic Health Record (EHR) patient message, or mailed letter] and direct provider referral. Provider interviews helped characterize the context of current lethal means screening and counseling practices within the health system, logistical considerations, and acceptability of L2L.

Population-based invitation messages to visit L2L emphasized anonymity and were co-designed with input from clinicians, public health researchers, and members of the Colorado Firearm Safety Coalition. Six combinations of initial + reminder messages (e.g., email + text; email + mail) were tested. Letter, email or EHR message were used for initial invites, but never text—due to length restrictions. Combinations of different methods were tested on the hypothesis that people will have varying response propensities to different methods.

For the provider referral pilot, nine mental health providers agreed to test L2L with patients for 6 weeks. Patients were selected for L2L referral from clinical judgment of the patient's suicide risk (not just the PHQ-9). Providers had a 1-h orientation to L2L with discussion and agreement on the best referral process. They were provided with scripting templates for introducing L2L. Collaboratively, a goal was set of each provider to refer 10 patients over a 6-week period (providers felt this was easily achievable at the outset). A follow-up discussion with providers identified barriers and facilitators to referral. All study procedures were approved by the Kaiser Permanente Institutional Review Board.

### Measures

#### Web analytics (Uptake)

Website visit rates assessed uptake of L2L for each outreach method using separate URLs (e.g., EHR + text, mail + text). This allowed tracking of visits associated with different outreach approaches but maintained patient anonymity. Text messages were only used as reminders, but never as initial messages due to length restrictions.

#### Patient survey (Storage behavior and acceptability – see Appendix A)

All patients received an anonymous survey to evaluate acceptability with L2L and safe storage behavior 4 weeks after their first invitation to visit L2L. Items assessed uptake (visits to website) and satisfaction with L2L, lethal means storage behavior (i.e., firearms and medications), and whether patients received lethal means counseling from medical or mental health providers. Stage of Change theory (25) informed survey questions for storage behavior by assessing pre-contemplative beliefs about storage, whether the patient was thinking about changing storage behavior, planning changes, or had taken action to change storage behavior. Survey development was undertaken in phases with an initial testing phase among non-study research colleagues and community members of the Colorado Firearm Safety Coalition (26). Part way through we amended some of the branching logic to capture whether L2L was influential towards patients considering or planning storage changes.

#### Provider interviews

The goal of the interviews was to characterize the context of current lethal means screening and counseling practices within the health system, logistical considerations and acceptability of L2L. Eligible providers were mental health therapists, psychiatrists, and primary care providers who responded to email invitations. An invitation email to participate in a 30–45-min interview was sent to a convenience sample of 13 mental health and 10 primary care providers. Interviews were held *via* video conference (Microsoft Teams) and followed an interview guide that assessed current lethal means safety counseling practices, barriers and facilitators for delivering lethal means counseling, and whether and how L2L could be a useful tool for integration either during visits or separately using population-based outreach methods. All interviewees provided informed consent and there was no incentive.

### Analysis

#### Reach

Defined as all eligible adult patients who endorsed suicide ideation on the PHQ-9 for the population-based approach. Patients were outreached in batches to evaluate the impact of different outreach methods. For the provider-referral approach, reach included all patients who were referred by providers.

#### Uptake

Uptake was defined by the number of unique IP addresses that visited each unique URL divided by the number of patients sent a message for that batch (outreach method). Email, mail, or EHR messages that bounced back were not included in the denominator. A logistic regression model determined differences in response rates between outreach methods.

#### Patient survey

Descriptive frequencies were calculated for each survey question based on the number of patients who answered that question. Patients who skipped questions or answered “prefer not to answer” were included in the denominator, but those who were never presented with the question because of branching logic or early discontinuation were not counted.

#### Provider interviews

Interviews were audio recorded. Two coders (who conducted the interviews) independently listened to the recordings and noted themes within the apriori topics from the interview guide. The coders then met and shared their ideas and resolved any discrepancies in interpretation. Themes were summarized in a table by the apriori topics and sent to each interviewee for member checking (24). Interviewees provided minor edits to the interpretations *via* email response.

## Results

Table 1 details patient and clinical characteristics of the sample. The patient sample (*N* = 2,931) was predominantly female (65%) with moderate to severe depression reported on the PHQ-9 (mean total score = 15.6).

**Table 1:** Patient characteristics (*N* = 2,931).

	*n*	Percent
**Age**		
18–24	602	20.5
25–44	1,299	44.3
45–64	761	26.0
65–89	264	9.0
90+	5	0.2
**Sex assigned at birth**		
Female	1,911	65.2
Male	1,020	34.8
**Race and ethnicity**		
American Indian and Alaska Native	17	0.6
Asian	66	2.3
Black	138	4.7
Hispanic	456	15.6
Native Hawaiian	5	0.2
Other	131	4.5
Unknown	215	7.3
White	1,903	64.9
**PHQ9 depression total score (0–27)**		
0–4 (minimal)	63	2.1
5–9 (low)	446	15.2
10–14 (moderate)	737	25.1
15–19 (moderate/severe)	873	29.8
20–27 (severe)	789	26.9
**PHQ9 suicide ideation item (9) score**		
Several days	2,096	71.5
More than half the days	511	17.4
Nearly everyday	324	11.1
Total	2,931	100.0

There were 23 patients with missing total PHQ-9 scores, but who had item-9 scores.

### Website visits

Population-based outreach messages were sent to 2,729 of 2,931 adults identified *via* the EHR; 202 patients (6.8%) were excluded because they were unreachable (i.e., no email address, no mobile number, returned mail). Table 2 illustrates the response rates for each outreach method tested. EHR messages plus two email reminders resulted in the highest L2L visitation rates of 11% (52/491) (OR = 13.7 (4.9–38.2) compared to mailed letter plus text reminder (REF) with the lowest L2L visitation rate (4/480; <1%).

**Table 2:** Lock to Live visit and survey completion rates by outreach method.

Message type	% Visited L2L website	Total patients	Odds ratio	95% CI
EHR message with 1 mail reminder (Batch 1)	6	468	7.2	2.5–20.7
EHR message with 1 text reminder (Batch 2 and 5)	7	904	9	3.3–25
Standard mail with 1 text reminder (Batch 3)	<1	456	REF	REF
Email with 1 mail reminder (Batch 4)	1	429	1.1	0.3–4.3
EHR message with 2 email reminders (Batch 6)	11	472	13.7	4.9–38.2

Logistic regression (likelihood ratio = 80.5, *p* < 0.0001). The invitation message was updated in batch 5 and 6 to de-emphasize suicide risk, add in more caring language, and add emphasis to general home safety. EHR message with 1 text reminder was tested twice to determine impact before (6%) and after COVID (8%).

In the 6-week provider referral pilot, three of the nine outpatient providers referred a total of 14 patients, with a 100% L2L visit rate. One patient (out of 14) had previously received an invitation *via* population-based outreach. In follow-up discussions, providers identified barriers to referral including perceived need for real-time EHR reminders about L2L availability, lack of visit time to bring up L2L, patients declining to learn more about the program, and perception that L2L is better suited for patients with moderate to severe suicide-risk.

### Patient survey

Of the 326 patients who started the survey, 44 (13.5%) didn't answer any questions, 114 (35.5%) answered some questions, and 168 (51%) completed all questions. Survey completion varied by outreach method (Table 2). Table 3 reports the satisfaction with L2L among survey respondents who reported visiting. Most indicated they would prefer to receive L2L through EHR messages (78%), followed by email (41%), provider (40%), poster in clinic (32%), text (26%), and mail (15%). Of the 286 patients who completed part/full survey, 46 (16%) indicated that a medical or mental health provider had discussed firearm safety and 34 (12%) discussed medication safety at a recent visit.

**Table 3:** Lock to Live satisfaction (*N* = 73).

Factor	Proportion reporting “Strongly Agree” or “Agree”
Easy to access from your device	70 (96%)
Easy to use	70 (96%)
Explained benefits, barriers, storage options well	70 (96%)
Found cost information useful	53 (73%)
Found safe storage information useful	68 (93%)
Storage options were too expensive	25 (34%)
Important that no personal info shared on website	67 (92%)
Had concerns about my privacy when answering questions on L2L	54 (74%)
Felt we respected privacy when sending L2L	63 (86%)

Only includes patients who indicated that they visited the Lock to Live website.

### Patient survey: Firearm storage section

Table 4 describes firearm storage beliefs and behaviors indicating 35% (*n* = 82/231) had a firearm. Of those with a firearm (*N* = 82), half (*N *= 41, 50%) did not endorse any secure storage behaviors such as locking devices or firearm safes. Of those who *endorsed at least 1 storage behavior*, one person indicated that L2L was influential (*N* = 41). Most said a friend, family member, or firearms safety class influenced their current storage behavior. Of those with access to a firearm *who did not endorse any storage behaviors*, 44% (*n* = 18/41) said they were thinking about firearm storage changes and 31% (*n* = 14/41) said they were planning changes. A later version of the survey indicated that 50% of those who had access to a firearm and were thinking about storage changes, thought L2L was influential to their considerations (*n* = 5/10, 50%).

**Table 4:** Patient reported firearm and medication storage beliefs and behaviors.

Factor	Level	Value
Important store firearms away from person at risk for suicide	Strongly agree/agree	215 (93%)
Important store firearm locked-up regardless whether someone in house has suicide risk	Strongly agree/agree	213 (92%)
Important store firearm unloaded regardless suicide risk in home	Strongly agree/agree	213 (92%)
Important to limit access to medications to a person at risk of suicide	Strongly agree/agree	209 (90%)
Patient has unsecured firearm access	Yes	41 (18%)
	No	186 (81%)
	Prefer not to answer	4 (1.7%)
Firearm storage behavior (among those who said that the patient cannot access a firearm)	Do not own firearm	149 (80.1%)
	Firearm is locked up	18 (9.7%)
	Firearm has locking device	3 (1.6%)
	Firearm moved out of home	4 (2.2%)
	Firearm is unloaded	10 (5.4%)
	Unsure where firearm is located (patient does not have control of firearm)	3 (1.6%)
	Prefer not to answer	3 (1.6%)
Medication access	Yes, all medications are accessible	180 (73%)
	Yes, some medications are accessible	22 (9%)
	No, medications are not accessible	19 (8%)
	Prefer not to answer	20 (8%)
	I’m not sure	7 (3%)
Med storage current (among those who endorsed any access restriction)	Meds locked up	26 (38%)
	Meds removed	3 (4%)
	Doc reduced meds available	3 (4%)
	Fam manages meds	10 (15%)
	Unused meds disposed	21 (31%)
	Prefer not to answer	19 (28%)
Med barriers	No one I can trust	11 (6%)
	Don’t believe in safe storage	34 (19%)
	No one to help manage meds	12 (7%)
	Don’t need med safety now	122 (70%)
	None	26 (15%)
	Prefer not to answer	6 (3%)
Themes from open ended question on barriers to medication storage (84 responses)	I’m not at risk for suicide, or am not suicidal now	17
	My medications aren't dangerous	13
	Medications wouldn't be my method of choice	6
	Locking up medications is generally unnecessary, no explanation given	7
	I’m already taking some precautions, or I plan to	9
	I live alone; no one can help me, or I can't lock them up from myself	11
	This would be inconvenient for myself or others, or I would forget to take them	15
	Locking up medications won't prevent suicide	2
	I’m not sure how to go about safe storage	4
	I’m not at risk for suicide, or am not suicidal now	17

The first firearm question asked if the patient has access to an unsecured firearm. If they said Yes, then we didn't ask them the 2nd question (41 patients said yes). If they said No, we then asked how the firearm was stored (this implicitly assumes that they have a firearm to help normalize this behavior among owners), but we offer an option for “do not own a firearm.” For the 2nd question a different 41 patients endorsed one of the firearm storage options—indicating there is a firearm in their home, but it is stored safely. Therefore 82 patients had a firearm, and ½ (*n* = 41) of these used secured storage.

### Patient survey: Medication storage

Table 4 describes medication storage behaviors. Of the 248 who answered questions in this section, 181 (73%) indicated that all medications are accessible. *Of those with medications accessible*, 36 (27%) were thinking about changes and of those, 57% (20) were planning changes. Disposing of old medications (*N* = 36, 73%) and medication lockbox (*N* = 23, 47%) were cited as the most common planned behaviors compared to family/friend managing meds (*N* = 12, 24%) and receiving smaller prescription amounts (*N* = 1, 2%). Of those who had medications stored inaccessibly, L2L was cited as influential in 4 cases (6%). In later batches, where we assessed influences on decision making for those considering changes, L2L was cited as the most influential factor, more so than providers, friends/family or other influences. Of the 268 patients who completed, 84 (31%) provided qualitative responses to the open-ended question about barriers to medication storage (Table 4).

### Provider interviews

Of the 23 providers invited, 16 responded and were interviewed: 5 primary care physicians, 3 psychiatry physicians, 3 behavioral health crisis clinicians, 3 behavioral health outpatient therapists, and 2 primary-care based psychotherapists.

#### Current lethal means counseling policies and practices

Providers were split evenly on whether they felt that patients are forthcoming when discussing access to firearms or medications. Most felt that they could handle resistance to firearm discussions by emphasizing their interest in the patient's safety and providing reassurance that firearm removal was temporary until suicidal thoughts have passed. There was disagreement on whether there was a standard operating procedure within the health system to ask all patients with suicide risk about firearm and medication access. Some mentioned that they ask all patients at intake visits but may not ask again if the initial assessment for access is negative. Providers noted that there are quality metrics for depression and suicide screening within the health system, but none for lethal means assessment.

“*We don't necessarily ask at every visit, but hopefully we ask in every first visit. If it's documented previously that they don't have access, we don't ask in every follow-up visit*.”

Providers said they often problem solve about how to limit access and ask if there are family or friends who can hold the lethal means temporarily. For the highest risk patients, this may involve reaching out to the patient's spouse, other family member or a friend. If the patient has a history of suicide risk or attempts, but isn't currently reporting ideation, safe storage options are recommended that might be necessary during higher-risk times.

“*We will problem-solve around how to put space between the patient and whatever lethal means are identified as accessible. We talk about giving firearms to the police, neighbor, etc. It seems like patients are honest about restricting access to lethal means; if they're not willing to restrict access, they're pretty quick to say so. For patients who don't endorse suicide ideation, we still recommend that they keep firearms secure, especially while going through that episode of depression*.”

#### Strategies to inform Lock to Live uptake among patients and providers

Providers said that it was important to highlight the privacy and anonymity of L2L. However, it was emphasized that it would be better not to mention the reason for outreach was their endorsement of suicidal thoughts on the PHQ-9 screening tool. Providers described how patients may be more suspicious of firearm safety information sent *via* email that was connected to their responses to a suicide screener unless it came directly from a trusted clinical provider (even though it came from the integrated health system).

“*I’m not sure it's feasible, but this would be good to send to everyone. There are people out there who aren't getting help; this program might be helpful to them. It would be beneficial to visit the site in-person with the provider. That would eliminate the concern about someone else having accessed their data. A provider can encourage them to participate, even just by giving them a card with the website link*.”

#### Provider perceptions of Lock to Live usefulness and patient acceptability

Nearly all the providers interviewed felt that L2L was a great resource that provided storage options they didn't know about (such as disassembling a firearm). In general, providers felt that patients would react positively to L2L, that it could promote behavior change and prompt conversations between patients and their care providers. An important consideration was that patients will have to be receptive to lethal means safety steps (beyond pre-contemplative stage of change) before accepting the tool. Many providers felt comfortable addressing patient resistance to the lethal means conversation but were less knowledgeable of different types of storage options.

“*I think L2L could promote behavior change and prompt discussion with providers; it takes away some stigma. Some of my colleagues are uncomfortable having these conversations, and L2L could be helpful in that regard*.”

## Discussion

We found that population-based outreach using the Lock to Live (L2L) web-based decision aid for safe storage of lethal means in those with suicide risk reached many patients who had not previously discussed lethal means safety with providers. While uptake to population-based approaches was low (11%) in comparison to provider-referral (100%), most of the low-risk patients outreached using population-based methods had not been previously counseled on lethal means safety per survey report. The absolute number who visited L2L in a 6-week time was 52 for population-based with the best method versus 14 for provider referral. Every patient referred by providers visited L2L, but without more organizational supports (e.g., reminders, quality metrics, more intensive training), reach will remain low. We learned a great deal from patients and providers about ways to improve uptake of L2L for both self-directed population-based methods and provider referral as part of lethal means counseling.

We saw that sending invitations securely through the electronic medical record with two email reminders had the highest L2L visit rate of 11%. EHR messages were the most preferred outreach method based on patient survey feedback and website-usage data. Low-response may be due to stigma often associated with seeking care for mental health conditions (27). The response rate was comparable to other population-based outreach efforts for sensitive issues, such as chlamydia screening (28), that have between 15% and 20% response.

Follow-up discussion with providers from the referral pilot illustrated that referral only felt appropriate for patients they assessed as moderate to high risk (e.g., *via* clinical assessment). Past studies have shown that providers are concerned that lethal means counseling could have a negative impact on the therapeutic relationship (10–12, 29)—although we did not hear that from providers in our study. Providers identified barriers including a lack of reminders, time restrictions, unclear protocols for lethal means access assessment and counseling, and no quality metrics to bring about accountability.

Our findings highlighted the need for future lethal means safety approaches to incorporate stages of change to promote secure lethal means storage. We observed that 18% reported access to an unsecured firearm, with 44% of these thinking about changing storage (contemplative) and 50% not considering changes (pre-contemplative). Behavior change interventions that don't incorporate patients readiness for change may be less likely to succeed (25). Provider interviews were consistent with this idea indicating that it may take more than one “nudge” to get patients to consider safe storage. Population-based outreach may be less effective to engage patients in a lethal means safety tool than provider-delivered intervention, but still valuable to move someone closer to behavior change over time. Combining these two methods could bring the most impact, particularly if the low-rate of provider-delivered counseling were improved through the suggestions offered here which were: EHR reminder, provider training, quality metrics with accountability, a clearly communicated lethal means screening/counseling policy, and strong organizational leadership support.

Future studies should not only measure whether someone with suicide risk has access to a firearm or medication, but whether the current level of suicide risk is consistent with the current storage behavior. Patient survey and provider interview feedback indicated that it may be important to approach changes in firearm storage using a risk-stratification—especially in individuals with low or intermittent suicide risk. Instead of static goals about safe storage, different storage options from less to more inaccessible may be considered based on current risk. Patients with protection weapons (36% of firearm owner respondents) may be more amenable to the idea of temporary off-site storage only during high-risk times, a strategy used by the Gun Shop project (30). For medication storage, 70% indicated that they don't need medication safety at this time, further illustrating the need to consider risk and timing with lethal means safety.

It is important to note limitations to our study that may impact generalizability to other populations. There are many patients who likely didn't respond to the survey or visit L2L due to uneasiness about privacy, even though both were anonymous to address this concern. The invitation message came from the health system and many are skeptical of sharing firearm ownership with medical establishments (10–12, 29). Response rates would likely be improved from community-based surveys or from organizations within the firearm community. However, it would be harder to identify those with suicide risk outside of an integrated health system that does routine screening. Another reason for non-response may be severity of depression symptoms (e.g., lack of motivation) since reported symptom severity was high (56% had total score PHQ9 > 15). One option would be to survey patients later after they have completed a course of treatment and experienced symptom improvement, but this may not be representative of patients’ receptiveness during times of suicide risk when lethal means safety is important. Since our sample was limited by those seeking mental health care services within an integrated healthcare system who reported suicide ideation, which were majority female, we may have missed men who are much higher risk of firearm suicide (31). Future studies of firearm safety should consider using alternative ways to identify patients at risk for firearm suicide such as predictive models (32). Finally, while we tested the text and email messages with common providers including AT&T and Verizon for text message and Gmail (google) for emails, there is a possibility that spam-blocking software prevented patients from receiving text or email messages. However, this would not impact the EHR messages. COVID-19 contextual factors must be considered for all research that was ongoing during this time. The pandemic may have detrimentally impacted providers’ ability to incorporate a new process that included referral to L2L. Conversely, increased number of new firearm owners during this time (33) may have fueled increased interest in firearm safety among patients and providers. Finally, our tracking methods did not allow us to measure how long patients spent on the L2L tool to understand whether they spent sufficient time to comprehend the content.

The goal of the current project was to assess feasibility (reach and uptake) and acceptability (patient survey and provider interviews) of outreach methods to the L2L web-based decision aid. We found that population-based approaches have higher reach and smaller uptake compared to provider referral, but the absolute number reached through population-based approaches was higher. Expanding provider-delivered lethal means safety interventions to all patients reporting suicide risk will require an EHR reminder, provider training, quality metrics with accountability, a clearly communicated lethal means screening/counseling policy, and strong organizational leadership support. Population-based outreach of lethal means safety is a viable low-cost option and may be particularly important tool as a primer to discussion of firearm access with providers.

## Data Availability

The raw data supporting the conclusions of this article will be made available by the authors, without undue reservation.

## Appendix A

### Sample Survey Questions

#### Definitions (Appear at the top of each page)

Medications: refers to any medications in the home, including prescription and over-the-counter medications, regardless of who owns or uses them.

Firearm: refers to any firearms in the home, including hand guns and long guns (such as rifles, shotguns, submachine guns, etc.), regardless of who owns the gun or its working condition.

Provider: generally refers to any health or mental health professional. This includes medical doctors (MD) or doctors of osteopathy (DO) in any medical specialty including primary care, endocrinology, cardiology, etc. A provider may also be a psychiatrist, psychologist, mental health therapist, social worker, nurse practitioner (NP) or physician's assistant (PA).

### Survey introduction

The survey should take less than 10 min to complete. At the end of the survey, you will have the chance to enter a drawing for a $100 Amazon gift card. **This survey is anonymous, which means we cannot link your answers back to you**. We appreciate your time! There are no right or wrong answers.

Before beginning, please tell us a few things to help us understand the entire group of people responding to this survey.

Which category below includes your age?

- 18–24 years old
- 25–44 years old
- 45–64 years old
- 65–89 years old
- Prefer not to answer

What gender do you identify with? Choose all that apply.

- Female
- Male
- Transgender man
- Transgender woman
- Other (please specify)
- Prefer not to answer

What is your race? Choose all that apply.

- American Indian
- Asian
- Black
- Native Hawaiian
- White
- Other
- Unknown
- Prefer not to answer

Are you of Hispanic, Latino, or Spanish origin (ethnicity)?

- Yes
- No
- Unknown
- Prefer not to answer

What is the highest level of school you have completed?

- Some high school
- High school graduate or GED equivalent Some college
- Trade/technical training
- Bachelor’s degree or higher
- Prefer not to answer

#### Instructions

This next set of questions is about how medications and firearms are currently stored in your home.

How much do you agree or disagree with the following statements? (Precontemplation)

Strongly agree: Strongly Disagree (4-point likert scale)

- It is important to securely store medications when I or someone in my household has had recent thoughts of suicide.
- It is important to securely store other potentially dangerous household items (such as knives, scissors, razor blades, ropes, or chemicals) when I or someone in my household has had recent thoughts of suicide.
- It is important to securely store firearms when I or someone in my household has had recent thoughts of suicide.
- It is always important to store firearms securely in a locked space, or with a cable/trigger lock, regardless of suicide risk for someone in the home.
- It is always important to securely store firearms unloaded regardless of suicide risk for someone in the home.

B6.) Are there any restrictions to medication access in your home for you or other members of your household? Choose the best option. B6

• Yes
• No
• I’m not sure
• Prefer Not to Answer

B6a.) Please choose the options that best describes medication storage in your home. (Check all that apply)

• Medications are locked up or secured and cannot be easily accessed
• Medications have been removed from the home
• A doctor has reduced the number of pills available for pick up at one time
• A family member or friend is now managing medications
• Unused medications have been thrown away
• Prefer not to answer

B6d.) Have you started thinking about ways to store medications differently (even if you haven't made any changes yet)? (Yes, No, Prefer not to answer)

B6e.) Are you planning to change how medications are stored in your home, such as finding or ordering a secure storage device, setting aside time to collect all medications, setting up time to talk with a trusted friend or family member about helping with medication management (even if you haven't made any changes yet)? (Yes, No, Prefer not to answer)

If Yes….

B6e1.) Have you thought about any of the following changes? Check all that apply.

○ Gathering medications and putting them in a locked cabinet, safe or lockbox.
○ Throwing away (or getting rid) of old or unused medications
○ Asking a trusted family member or friend to help manage medications
○ Asking for smaller amounts of prescription medications from medical providers
○ Other (please describe)
○ Prefer Not to Answer

REMINDER: Information about your participation on the website and responses to the survey are completely anonymous. We cannot link this survey back to you in any way.

B7) Are you able to access a firearm(Yes, No, I’m Not Sure, Prefer Not to Answer). Choose the best option.

B7a.) Please choose the options that best describe your firearm access (Check all that apply)

- There are no firearms in my home or anywhere else where I regularly spend time (e.g., friend or family's house, workplace).
- Firearms are locked-up (gun safe, cabinet, etc.) and cannot be accessed.
- Firearms have a locking device and cannot be accessed.
- Firearms have been moved to a different location outside the home that cannot be accessed.
- Firearms are unloaded and ammunition cannot be accessed.
- I am not sure where the firearms are located in my home, so I do not know how they are stored.
- Prefer Not to Answer

B7c.) Have you started to think about ways to store firearms differently (even if you haven't made any changes yet)? (Yes, No, Prefer not to answer)

B7d.) Have you started planning changes to how firearms are stored in your home, such as: talking with a friend/family about storing your firearms, finding or ordering a new locking device or setting aside time to unload firearms (even if you haven't made any changes yet)? (Yes, No, Prefer not to answer)

If Yes….

B7e1.) Have you thought about any of the following changes? Check all that apply.

○ I have thought about purchasing locking devices
○ I have thought about a safe or lockbox
○ I have thought about temporarily giving my firearm to a trusted family member or friend for safekeeping
○ I have thought about temporarily storing my firearms at a gun store, range, or other private business
○ I have thought about temporarily storing my firearms at a law enforcement agency
○ Other (please describe)
○ Prefer Not to Answer

B7e2.) Did any of the following help you to think about different ways firearms could be stored in the home? (Check all that apply)

○ Information from the *Lock to Live* website
○ Talking with a Kaiser Permanente provider
○ Talking with a provider outside of Kaiser Permanente
○ Talking with a friend or family member
○ Other (please describe)
○ None of the above
○ Prefer not to answer
